# Simultaneous development of Kawasaki disease following acute human adenovirus infection in monozygotic twins: A case report

**DOI:** 10.1186/s12969-017-0169-x

**Published:** 2017-05-16

**Authors:** Sayaka Fukuda, Shuichi Ito, Maya Fujiwara, Jun Abe, Nozomu Hanaoka, Tsuguto Fujimoto, Hiroshi Katsumori

**Affiliations:** 1 Department of Pediatrics, Kawakita General Hospital, 1-7-3 Asagaya-kita, Suginami-ku, Tokyo Japan; 2 Department of Pediatrics, Graduate School of Medicine, Yokohama City University, 3-9 Fukuura, Kanazawa-ku, Yokohama Japan; 3 Department of Allergy and Immunology, National Center for Child Health and Development, 2-10-1 Okura, Setagaya-ku, Tokyo Japan; 4 Infectious Disease Surveillance Center, National Institute of Infectious Diseases, 1-23-1 Toyama, Shinjuku-ku, Tokyo Japan

**Keywords:** Kawasaki disease, Human adenovirus, Monozygotic twins, Pathogenesis, Genetic susceptibility

## Abstract

**Background:**

The etiology of Kawasaki disease (KD) remains unknown. However, many studies have suggested that specific genetic factors and/or some infectious agents underlie the onset of KD. Previous studies have suggested that human adenovirus (HAdV) is one of the triggering pathogens of KD. Here, we report monozygotic twin boys who sequentially developed KD in conjunction with acute HAdV type 3 (HAdV-3) infection.

**Case presentation:**

The patients were four-year-old monozygotic twin boys. The elder brother developed a high fever and was diagnosed with HAdV infection with an immunochromatographic kit for HAdV (IC-kit). He was transferred to our institute after persistent fever for 7 days. On admission, he already fulfilled all the diagnostic criteria for KD. His laboratory data were as follows: WBC, 9700/μl; CRP, 2.42 mg/dl; IFN-γ, 99.8 pg/ml; and TNF-α, 10.9 pg/ml. He received intravenous immunoglobulin (IVIG) and aspirin and responded well, with no coronary artery abnormalities. The younger brother, who was also IC-kit-positive, was hospitalized on the same day as his elder brother after persistent fever for 3 days. His data on admission were as follows: WBC, 12,600/μl; CRP, 5.54 mg/dl; IFN-γ, 105.0 pg/ml; and TNF-α, 33.6 pg/ml. Although he developed all of the typical KD symptoms by day 4, his fever subsided spontaneously on day 6 without IVIG or aspirin. However, he developed a dilation of the coronary artery in the region of the left circumflex artery bifurcation on day 10. His coronary artery dilation had resolved 3 months after onset. HAdV-3 DNA was detected with PCR in stool samples from both patients, and HAdV3 was isolated from the younger brother’s stool sample. Serum neutralizing antibodies to AdV3 were also significantly elevated in both patients, suggesting seroconversion.

**Conclusions:**

There have been few reports of the simultaneous development of KD in monozygotic twins. Notably, both twins had an acute HAdV-3 infection immediately before they developed KD. These cases strongly suggest that KD was triggered by HAdV-3 infection, and they indicate that specific immune responses to some pathogens (such as HAdV-3), arising from genetic susceptibility, play a critical role in the pathogenesis of KD.

## Background

Kawasaki disease (KD) is an acute systemic vasculitis syndrome, first reported by Dr. Tomisaku Kawasaki in 1967 [1]. Although its etiology remains unknown, specific genetic factors and/or some infectious agents may underlie the onset of KD [2, 3]. Previous studies have shown that human adenovirus (HAdV) might be one of the pathogens that triggers KD [4]. Here, we describe a case report of monozygotic twins who simultaneously developed KD after an acute HAdV type 3 (HAdV-3) infection, which was confirmed by PCR-sequencing, virus isolation, and seroconversion. Our case is the first report of KD associated with monozygotic twins who suffered a HAdV-3 infection, and it supports the hypothesized pathogenesis described above.

## Case presentation

The patients were four-year-old monozygotic twin boys who had previously been healthy and had no family history of KD.

### Case 1: Elder brother

**Fig. 1:**
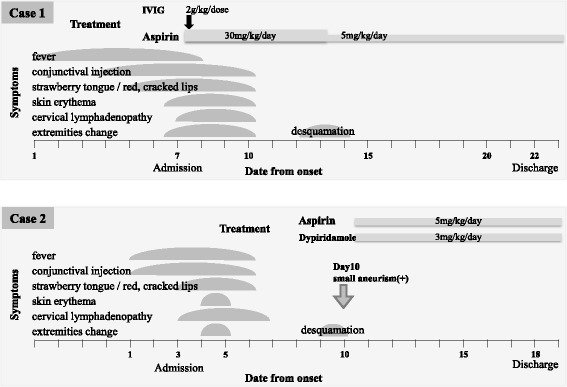
Clinical course of the patients

The elder brother developed a high fever on day 1 of his illness and subsequently developed redness of the eyes, red cracked lips, strawberry tongue, erythema, swollen red palms and soles, and cervical lymphadenopathy. He was admitted to our hospital on the seventh day after fever onset. On admission, he met all six diagnostic criteria for KD and was also diagnosed with HAdV infection with a rapid test for AdV (Imunoace^®^adeno, TAUNS Laboratories Inc. Shizuoka, Japan). His laboratory data were: white blood cells (WBC), 9700/μL (neutrophil sequestration, 66.0%); hematocrit, 38.8%; platelet count, 35.7 × 10^4^/μL; albumin, 4.1 g/dL; total bilirubin, 0.5 mg/dL; sodium, 130 mEq/L; aspartate aminotransferase (AST), 31 IU/L; alanine aminotransferase (ALT), 16 IU/L; and C-reactive protein, (CRP) 2.4 mg/dL. His serum cytokine profile was: granulocyte-colony stimulating factor (G-CSF), 384.0 pg/mL; interferon-γ (IFN-γ), 99.8 pg/mL; interleukin-6 (IL-6), 43.8 pg/mL; IL-8, 36.9 pg/mL; IL-18, 1200.6 pg/mL; tumor necrosis factor (TNF-α), 10.9 pg/mL; soluble tumor necrosis factor receptor 1 (sTNFR-1), 1106.9 pg/mL; and sTNFR-2, 10,013.4 pg/mL (Table 1). The patient was immediately treated with 2 g/kg intravenous immunoglobulin (IVIG) and aspirin. He responded well and achieved defervescence the next day, and all his symptoms disappeared promptly. Desquamation of the fingers was observed on day 12 of illness. He discharged on the 16th day of hospitalization (Fig. 1). A transthoracic echocardiography revealed no coronary artery abnormalities.

**Table 1:** Patient laboratory data on admission

		(normal range)	Case 1	Case 2
WBC	/μL		9700	12,600
Neut.	%		66.0	71.0
Htc.	%		38.8	36.8
Plt.	×10^4^/μL		35.7	39.2
Albumin	g/dL		4.14	4.33
Tbil.	mg/dL		0.5	0.6
Na	mEq/L		130.4	132.3
AST	IU/L		31	30
ALT	IU/L		16	13
CRP	mg/dL		2.42	5.54
G-CSF	pg/mL	(<100 pg/mL)	384.0	511.0
IFN-γ	pg/mL	(<30 pg/mL)	99.8	105.0
IL-6	pg/mL	(<20 pg/mL)	43.8	37.6
IL-8	pg/mL	(<30 pg/mL)	36.9	33.5
IL-18	pg/mL	(<500 pg/mL)	1200.6	580.4
TNFα	pg/mL	(<20 pg/mL)	10.9	33.6
sTNFR-1	pg/mL	(<500 pg/mL)	1106.9	736.4
sTNFR-2	pg/mL	(<5000 pg/mL)	10,013.4	8063.8

*WBC* white blood cells, *Neut*. neutrophil sequestration, *Htc*. hematocrit, *Plt*. platelet count, *Tbil*. total bilirubin, *Na* sodium, *AST* aspartate aminotransferase, *ALT* alanine aminotransferase, *CRP* C-reactive protein, *G-CSF* granulocyte colony-stimulating factor, *IFN*-γ interferon-γ, *IL*-6, -8, -18 interleukin-6, -8, -18, respectively, *TNF-α* tumor necrosis factor,

*sTNFR*-1, −-2 soluble tumor necrosis factor receptor 1, 2, respectively

### Case 2: Younger brother

The younger brother also presented with a high fever and redness of eyes 4 days after his elder brother’s symptom onset, and he then developed red cracked lips, strawberry tongue, and cervical lymphadenopathy on day 3. The patient was hospitalized on the same day as his elder brother after experiencing a persistent fever for 3 days, and a rapid test for HAdV was positive at this time. His laboratory data upon admission were: WBC, 12,600/μL (neutrophil sequestration, 65.1%); hematocrit, 36.8%; platelet count, 39.2 × 10^4^/μL; albumin, 4.3 g/dL; total bilirubin, 0.6 mg/dL; sodium, 132 mEq/L; AST, 30 IU/L; ALT, 13 IU/L; and CRP, 5.5 mg/dL. His serum cytokine levels were: G-CSF, 511.0 pg/mL; IFN-γ, 105.0 pg/mL; IL-6, 37.6 pg/mL; IL-8, 33.5 pg/mL; IL-18, 580.4 pg/mL; TNF-α, 33.6 pg/mL; sTNFR-1, 736.4 pg/mL; and sTNFR-2, 8063.8 pg/mL (Table 1). Unlike his brother, this patient met only three of the diagnostic criteria for KD upon admission, so we diagnosed him as having incomplete KD and did not treat him with IVIG or aspirin. On the day 5 of illness (2 days post-admission), the patient displayed all six symptoms of KD, but his fever spontaneously resolved on day 6 of illness without treatment (the patient received neither IVIG nor aspirin). After defervescence, all symptoms soon disappeared, and his laboratory data normalized. His CRP was 2.74 mg/dL on day 5 of illness and 0.22 mg/dL on day 10 of illness. On day 9 of illness, finger desquamation was observed. Unlike his brother, this patient’s echocardiography revealed a dilation of the left circumflex artery on day 10 of illness. We commenced treatment with 5 mg/kg/day of aspirin and 3 mg/kg/day of dipyridamole. He was discharged on the 16th day of hospitalization (Fig. 1). The maximum diameter of the coronary artery abnormality reached 3.9 mm (Z score, 4.7), but the coronary artery abnormality resolved within 3 months of onset.

Fecal samples were collected from both patients during the acute phase of the disease. These samples were tested by PCR assays, and HAdV-3 genomes were detected in the samples from both patients. Additionally, HAdV-3 was directly isolated from the younger brother’s fecal sample. Both patients were negative for serum neutralizing antibodies against HAdV-3 upon admission, but these antibodies were significantly elevated in both patients 2 weeks after their admission, suggesting that they underwent seroconversion. The patients’ father and elder sister also developed clinical symptoms consistent with acute HAdV infection around the time that the twins were infected.

## Discussion

Here, we have presented a case of 4-year-old monozygotic twins who sequentially developed KD in conjunction with HAdV-3 infection. There have been few case reports of monozygotic twins who developed KD simultaneously, and only two of these reports identified the trigger of KD (Table 2) [5–10].

**Table 2:** Previous reports of Kawasaki disease in monozygotic twins

Author (country)			Age (month)	Sex	Interval of onset	Twin A	Twin B	Trigger
						(Symptoms of KD/CAA^a^)		
Fink HW.	1985	(USA)	10	female	same day	6/−	6/−	unknown
Hoshino K, et al.	1990	(Japan)	16	female	same day	5/+	5/−	unknown
Kuijpers TW, et al.	2000	(Netherlands)	29	female	3 days	6/−^a^	5/−	measles virus infection
Ide T, et al.	2007	(Japan)	13	female	1 day	6/−	4/−	measles-rubella vaccine
Kottek A, et al.	2011	(USA)	18	male	2 days	5/−	3/−	unknown
Zhang X, et al.	2013	(China)	2	female	same day	4 / +	4/−	unknown

*CAA* coronary artery abnormalities, ^a^ transient dilation

It has recently been presumed that the etiology of KD combines genetic susceptibility and specific infection, both of which are essential for KD development. Patients with KD seem to have some genetic predisposition, and ethnic differences in the morbidity and familial aggregation of KD have been reported [11]. Some functional single-nucleotide polymorphisms (SNPs) of genes such as inositol 1,4,5-trisphosphate 3-kinase C (*ITPKC*) and caspase 3 (*CAPS3*) significantly increase susceptibility to KD [2, 12, 13].

HAdV, group A streptococcus, *Staphylococcus*, *Bacillus cereus*, and *Yersinia* have all been reported as triggering pathogens of KD. In general, KD most commonly develops in infants, toddlers, and young children; adult patients are rarely reported [14]. Most children get infected with common pathogens, such as HAdV, during early childhood, so if these pathogens can trigger KD, this may partially explain the higher KD prevalence in children than that in adults.

HAdV infection itself could be one of the differential diagnoses of KD. HAdV infection also shows KD-like symptoms such as conjunctival injection, red cracked lips, and cervical lymphadenopathy. However, skin erythema and swollen red palms and soles followed by desquamation are distinctive features of KD. Additionally, a coronary lesion can allow the definitive diagnosis of KD. In our case, the younger brother’s echocardiography revealed a dilation of the left circumflex artery, so we definitively diagnosed him with KD.

Several previous reports show that HAdV was detected by PCR in samples from the respiratory tract of patients with KD [15–17]. However, it is difficult to distinguish between latent and acute HAdV infection by this method. Coincidental isolation of HAdV by PCR may also occur in some patients with KD. In our case, monozygotic twins simultaneously developed KD after acute HAdV-3 infection. HAdV-3 was detected in stool samples from both patients by PCR, and HAdV3 was directly isolated from the younger brother’s stool sample. Additionally, serum neutralizing antibody to HAdV-3 was significantly elevated in both patients 2 weeks after admission, compared with undetectable levels upon admission. This sero-conversion suggests acute infection. Furthermore, the elevated serum IFN-γ and IL-18 levels observed in these patients might also reflect a systemic inflammatory reaction against an acute viral infection. Therefore, we hypothesize that HAdV infection in children with a genetic susceptibility to KD may abnormally stimulate their innate immunity and evoke a cytokine storm leading to the development of KD. As acute infection can trigger KD, KD may have self-limiting and acute-onset features.

## Conclusion

Our report contributes further evidence that a specific response to a pathogen such as HAdV, combined with genetic susceptibility, plays an essential role in the development of KD.

## Abbreviations

ALT: Alanine aminotransferase
AST: Aspartate aminotransferase
CRP: C-reactive protein
G-CSF: Granulocyte colony-stimulating factor
HAdV: Human adenovirus
HAdV-3: Human adenovirus type 3
IFN-γ: Interferon-γ
IL-18: Interleukin-18
IL-6: Interleukin-6
IL-8: Interleukin-8
IVIG: Intravenous immunoglobulin
KD: Kawasaki disease
sTNFR-1: Soluble tumor necrosis factor receptor 1
sTNFR-2: Soluble tumor necrosis factor receptor 2
TNF-α: Tumor necrosis factor α
WBC: White blood cells
